# Artificially Sweetened Beverages and Health Outcomes: An Umbrella Review

**DOI:** 10.1016/j.advnut.2023.05.010

**Published:** 2023-05-13

**Authors:** Cristina Diaz, Leandro F.M. Rezende, Angelo Sabag, Dong Hoon Lee, Gerson Ferrari, Edward L. Giovannucci, Juan Pablo Rey-Lopez

**Affiliations:** 1 Faculty of Health Sciences, International University of Valencia (VIU), Valencia, Spain; 2 Department of Preventive Medicine, Escola Paulista de Medicina, Universidade Federal de São Paulo, São Paulo, Brazil; 3 NICM Health Research Institute, Western Sydney University, Westmead, New South Wales, Australia; 4 Discipline of Exercise and Sport Science, Faculty of Medicine and Health, The University of Sydney, Sydney, New South Wales, Australia; 5 Department of Nutrition, Harvard T.H. Chan School of Public Health, Boston, MA, United States; 6 Department of Sport Industry Studies, Yonsei University, Seoul, Republic of Korea; 7 Universidad de Santiago de Chile (USACH), Escuela de Ciencias de la Actividad Física, el Deporte y la Salud, Santiago, Chile; 8 Department of Epidemiology, Harvard T.H. Chan School Public Health, Boston, MA, United States; 9 Facultad de Deporte, UCAM. Universidad Católica de Murcia, Murcia, Spain

**Keywords:** acefulsame potassium, artificially sweetened beverages, microbiota, sucralose, umbrella review

## Abstract

The consumption of artificially sweetened beverages (ASBs) is increasing in some countries. However, some meta-analyses have found that habitual consumers of ASBs (compared with low or no consumption) had an increased risk on some health outcomes. We performed an umbrella review of meta-analyses to grade the credibility of the evidence of claimed observational associations between ASBs and health outcomes. Data were searched in Web of Science, Embase, and PubMed for systematic reviews published up to 25 May 2022, examining association between ASBs and any health outcomes. Certainty of the evidence for each health outcome was obtained based on statistical results of tests used in umbrella reviews. The AMSTAR-2 tool (16 items) was used to identify high-quality systematic reviews. Answers of each item were rated as yes, no, or partial yes (for a partial adherence to the standard). We included data from 11 meta-analyses with unique population, exposure, comparison group, outcome obtained from 7 systematic reviews (51 cohort studies and 4 case-control studies). ASBs were associated with higher risk of obesity, type 2 diabetes, all-cause mortality, hypertension, and cardiovascular disease incidence (supported by highly suggestive evidence). Evidence for other outcomes (colorectal cancer, pancreatic cancer, gastrointestinal cancer, cancer mortality, cardiovascular mortality, chronic kidney disease, coronary artery disease, and stroke) was weak. Results of the quality assessment of systematic reviews using AMSTAR-2 showed some notable deficiencies: unclear sources of funding of eligible studies and lack of predefined study protocols to guide authors. The consumption of ASBs was associated with a higher risk of obesity, type 2 diabetes, all-cause mortality, hypertension, and cardiovascular disease incidence. However, further cohort studies and clinical trials in humans are still needed to understand the impact of ASBs on health outcomes.

## Introduction

Artificially sweetened beverages (ASBs) are marketed as a replacement for sugar-sweetened beverages (SSBs). The food and beverage industry (or industry-funded studies) consider ASBs a healthy choice because they are sugar-free, with low or no calories. In United States, sales of regular soda decreased between 2006 and 2015 and sales of bottle water increased [1]. Health concerns related with sugary beverages consumption may favor the adoption of other healthier purchases among consumers.

However, despite the reassuring claims surrounding artificially sweetened products, there is growing evidence that the consumption of ASBs may not be totally healthy for humans. Experimental studies have found rapid changes in the gut microbiome in mice [2] and humans [2,3], which may play important roles in regulating metabolism, appetite, and fat storage. In some systematic reviews and meta-analyses of cohort studies, a high consumption of ASBs (compared with the lowest) was associated with a higher risk of all-cause mortality [[4], [5], [6]], CVD mortality [5,6], and cancer incidence [7]. In contrast, for cancer mortality, no evidence of harmful associations was found in 2 meta-analyses [5,6].

Umbrella reviews summarize the strength of associations reported in previous systematic reviews and meta-analyses. This new methodological approach aims to overcome the growing number of overlapping and conflicting reviews across an entire field [8,9].

Because of the existing conflicting results in recent reviews, we performed an umbrella review of systematic reviews and meta-analyses to evaluate the certainty of evidence of claimed observational associations between ASBs and health outcomes; and identify potential biases and inconsistencies. We also evaluated the methodological quality of systematic reviews and meta-analyses to guide future methodologists and authors in the design, analysis, and reporting of systematic reviews of ASBs and health outcomes.

## Methods

### Literature search

We searched Web of Science, Embase, and PubMed for systematic reviews published up to 25 May 2022, examining the association between ASBs and any health outcomes. One researcher (A.S.) was in charge of the first identification of the literature, once authors agreed on what keywords and searching databases were appropriate. The PRISMA 2020 guideline was used to describe the results of the literature search [10]. Supplemental Table 1 shows details of the search strategy used in the present umbrella review.

### Eligibility criteria

We selected systematic reviews (whether they performed meta-analyses or not) evaluating the association of ASBs and any health outcome; of cohort (among healthy populations at baseline) and case-control studies, and randomized controlled trials. When meta-analyses pooled cross-sectional studies, we excluded them because they provide a weak level of evidence by design. If meta-analyses included a mix of study designs (cohort, case-control, and cross-sectional), we specifically removed cross-sectional studies to reanalyze our meta-analyses. When systematic reviews included original studies whose exposure variable evaluated other sources of artificial sweeteners instead of ASBs (for example, sweeteners added to foods, or tabletop sweeteners), we excluded them. We also excluded systematic reviews that only pooled data of SSBs or energy drinks or juices in their analyses. Whenever >1 eligible systematic review addressed the association between ASBs and a particular health outcome, we selected the meta-analysis with the largest number of studies included. Narrative reviews and systematic reviews published as conference abstracts were also excluded.

Two researchers (C.D. and J.P.R.L.) independently screened the articles by title and abstract using the software Rayyan (https://www.rayyan.ai) and read full-text articles for the selection stage. A third author (A.S.) settled disagreements among researchers.

### Data extraction

Two researchers (C.D. and J.P.R.L.) independently extracted the following data: *1*) first author name and year of publication, *2*) population, exposure, comparison group, outcome (PECO) question, *3*) exposure variable, *4*) outcome variable, *5*) number of studies included in each systematic review and the number of studies included in the umbrella review, *6*) number of cohort studies, *7*) number of case-control studies, *8*) effect size (RR and 95% CI) of the fully adjusted model that compared the highest with the lowest ASBs intake, and *9*) funding source to conduct the systematic review. If responses were inconsistent between authors, they discussed again the original data to reach a consensus response.

### Identification of high-quality systematic reviews-AMSTAR-2

Two researchers (C.D. and J.P.R.L.) assessed the methodological quality of systematic reviews using the tool AMSTAR-2 [11]. This tool has 16 items to help researchers to identify high-quality systematic reviews: *1*) use of PECO elements in the description of aims and methods of the review, *2*) adherence to a well-developed study protocol, *3*) justification of the selection of study designs, *4*) use of a comprehensive literature search strategy, 5) study selection in duplicate, *6*) data extraction in duplicate, *7*) provide a list of excluded studies and justification, *8*) description of included studies in adequate detail, *9*) proper technique used to assess risk of bias of included studies, *10*) reporting of the sources of funding of selected studies, *11*) appropriate methods for statistical analyses of the meta-analysis, *12*) results of risk of bias assessments were considered in meta-analyses, 13) results of risk of bias assessments were discussed, *14*) the sources of statistical heterogeneity were discussed, and *15*) publication bias was assessed and discussed. Answers for each item were rated as yes, no, or partial yes (for a partial adherence to the standard).

### Statistical analyses

To estimate the average effect (RR and 95% CI) of each eligible systematic review and meta-analysis, we standardized the lowest reported ASBs intake as the reference group (compared with the highest intake) and performed a random-effects model. Results from a fixed-effect model were also provided, which are recommended when lack of heterogeneity among studies exist. To quantify inconsistency among studies or variability in the effects (statistical heterogeneity), we used *I*^2^. *I*^2^ describes the percentage of variability in effect estimates that is because of heterogeneity rather than sampling error. Prediction interval was calculated to consider the effects within 1 individual study setting, which may be different from the average effect [12]. Small study effects bias was assessed for each meta-analysis by the regression asymmetry test (Egger’s test) [13]. Egger’s test *P* < 0.10 and if the magnitude of association in the study with smaller standard error (largest study) of the meta-analysis was more conservative than the random-effects estimate of the meta-analysis. The excess significance test was calculated, which determines whether the expected number of studies (E) differs from the actual observed number of studies (O) with statistically significant results (*P* < 0.05) included in each meta-analysis [14]. Certainty of evidence was classified into 4 categories (strong, highly suggestive, suggestive, and weak) according to the following criteria: *P* value of the meta-analysis of a random-effects model, *P* value from the Egger’s regression asymmetry test (small study effects), *P* value of the excess of significance test using the largest study in a meta-analysis and number of cases. These criteria have been used in previous umbrella reviews in the literature [9]). All statistical analyses were analyzed using Stata version 16.

## Results

### Identification of systematic reviews of ASBs and health outcomes

**FIGURE 1:**
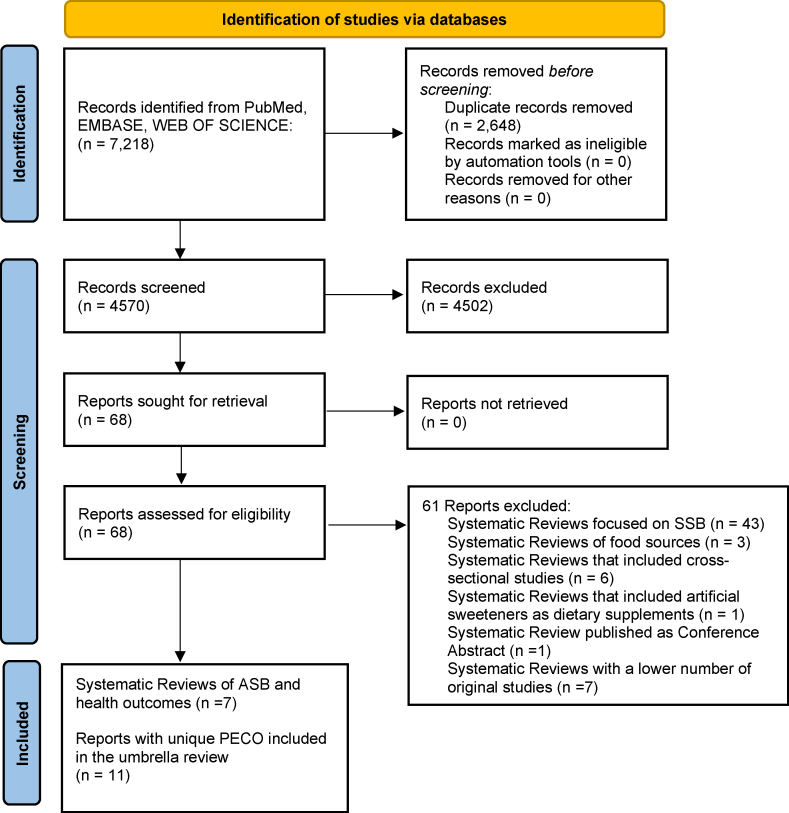
PRISMA 2020. Identification of systematic reviews of ASBs and health outcomes. ASB, artificially sweetened beverage.

Of 4570 records screened from the databases and after duplicates were removed (Figure 1), 11 reports with unique PECO were included in the umbrella review (obtained from 7 systematic reviews) [4,6,[15], [16], [17], [18], [19]].

Supplemental Table 2 shows descriptive data of the 11 selected reports of 7 unique systematic reviews. The outcome variables examined were: cardiovascular mortality [15], gastrointestinal cancer [16], colorectal cancer [16], chronic kidney disease [17], all-cause mortality [6], cancer mortality [6], obesity [4], type 2 diabetes [4], hypertension [4], CVD incidence [18], and pancreatic cancer [19]. The 11 selected reports had a total of 51 cohort studies and 4 case-control studies. In 5 reports from 4 systematic reviews [4,6,14,18], a higher intake of ASBs was associated with an increased risk of cardiovascular mortality, all-cause mortality, obesity, type 2 diabetes, hypertension, and CVD incidence. Authors declared the following sources of funding to perform their respective systematic reviews: no funding source [15], unreported information [16], hospitals [17,6], foundations [4], and national programs [18,19].

Supplemental Table 3 shows the original studies excluded (and reasons) of selected systematic reviews and meta-analyses. In some meta-analyses of ASBs, authors pooled original data of only SSBs (7 studies), or SSBs plus ASBs (11 studies), or SSBs plus ASBs plus juices (2 studies), the exposure included artificial sweeteners by supplements added to drinks from packets (8 studies), the exposure variable was poorly defined: ex. artificial sweeteners (unclear if they included food supplements plus beverages or not) (2 studies), the outcome variable included a nonspecific cancer site (1 study), the outcome variable was prediabetes (instead of type 2 diabetes) (1 study), and the exposure group was nonconsumers of ASBs (3 studies).

### Certainty of the evidence of the associations between ASB and health outcomes

Results of the umbrella review are shown in Table 1. Two additional health outcomes (CAD and stroke) were meta-analyzed and added to main results of the umbrella review from original studies pooled in the CVD incidence review [18]. Nine out of the 13 meta-analyses showed statistically significant associations (*P* < 0.05) in the random effect model. The effect sizes (RR) ranged between 1.13 (hypertension) and 2.28 (chronic kidney disease). When the *P* < 10^−6^ of the random effect model was used as a threshold, 4 associations remained statistically significant: all-cause mortality (RR, 1.19; 95% CI: 1.11, 1.27), obesity (RR, 1.55; 95% CI: 1.23, 1.96), type 2 diabetes (RR, 1.39; 95% CI: 1.22, 1.57), hypertension (RR, 1.13; 95% CI: 1.10, 1.16), and CVD incidence (RR, 1.23; 95% CI: 1.14, 1.32).

**TABLE 1:** Umbrella review meta-analyses (cohort and case-control studies) of artificially sweetened beverages and health outcomes

Health outcome	No. of studies	No. of cases/ total sample size	Random effects, RR (95% CI)	Random effects, *P* value	Fixed effects, RR (95% CI)	Fixed effects, *P* value	95% Prediction interval	I^2^ (*P* value)	Egger’s *P* value	Excess significance O/E (*P* value)	Largest study, RR (95% CI)
Cardiovascular mortality	3	20,599/783,382	1.29 (1.09, 1.54)	0.004	1.25 (1.16, 1.34)	0.000	0.16, 10.42	79.8 (0.01)	0.28	3/2.86 (0.710)	1.13 (1.02, 1.25)
Gastrointestinal cancer	8	5857/1,747,689	1.10 (0.95, 1.27)	0.191	1.10 (0.98, 1.23)	0.112	0.78, 1.55	33.2 (0.16)	0.90	1/1.37 (NA)	1.01 (0.77, 1.31)
Colorectal cancer	4	3316/744,742	1.00 (0.84, 1.20)	0.970	1.00 (0.87, 1.16)	0.985	0.55, 1.83	33.6 (0.21)	0.73	0/1.16 (NA)	1.01 (0.77, 1.31)
Chronic kidney disease	2	837/4188	2.28 (1.34, 3.88)	0.002	2.16 (1.48, 3.16)	0.000	-	17.5 (0.27)		2/2.00 (NA)	2.02 (1.36, 3.01)
All-cause mortality	6	84,729/988,807	1.19 (1.11, 1.27)	0.000	1.20 (1.16, 1.25)	0.000	0.97, 1.46	63.7 (0.02)	0.54	5/6.00 (NA)	1.21 (1.14, 1.29)
Cancer mortality	3	16,135/639,394	1.08 (0.92, 1.26)	0.328	1.12 (1.03, 1.21)	0.006	0.21, 5.57	62.1 (0.07)	0.13	1/2.66 (NA)	1.16 (1.04, 1.29)
Obesity	4	2087/11,168	1.55 (1.23, 1.96)	0.000	1.51 (1.28, 1.78)	0.000	0.67, 3.61	44.2 (0.15)	0.36	3/3.85 (NA)	1.59 (1.23, 2.07)
Type 2 diabetes	10	22,424/324,041	1.39 (1.22, 1.57)	0.000	1.24 (1.18, 1.30)	0.000	0.95, 2.02	74.2 (0.00)	0.03	7/4.60 (0.130)	1.10 (1.03, 1.19)
Hypertension	3	56,893/132,779	1.13 (1.10, 1.16)	0.000	1.13 (1.10, 1.16)	0.000	0.97, 1.32	0.0 (0.91)	0.99	1/1.74 (NA)	1.13 (1.10, 1.16)
CVD incidence	7	13,570/574,772	1.23 (1.14, 1.32)	0.000	1.23 (1.15, 1.31)	0.000	1.09, 1.39	7.7 (0.37)	0.05	4/4.65 (NA)	1.20 (1.09, 1.31)
CAD	3	9949/306,793	1.16 (1.05, 1.28)	0.002	1.15 (1.07, 1.23)	0.000	0.47, 2.84	34.4 (0.22)	0.49	2/2.52 (NA)	1.10 (1.00, 1.20)
Stroke	4	7149/308,298	1.24 (1.09, 1.42)	0.001	1.22 (1.12, 1.32)	0.000	0.81, 1.91	28.9 (0.24)	0.21	3/2.43 (0.560)	1.20 (1.09, 1.31)
Pancreatic cancer	5	2956/1,104,702	1.14 (0.93, 1.40)	0.215	1.15 (0.97, 1.37)	0.102	0.69, 1.88	25.7 (0.25)	0.24	0/3.57 (NA)	1.25 (0.94, 1.66)

Four out of the 13 meta-analyses had *I*^2^ < 30% (heterogeneity might not be important), 4 showed moderate heterogeneity *I*^2^ > 30%–50%, and 5 showed substantial or considerable heterogeneity *I*^2^ > 50%. In only 1 meta-analysis [18], prediction interval values excluded the null value (1.09–1.39). All meta-analyses showed small study effects bias, as the effect estimate of the largest study was more conservative compared with the summary random-effects estimate, and *P* values of the Egger test were >0.1.

Regarding the certainty of evidence (see results in Table 2), none of the associations examined were supported by strong evidence. The associations between ASBs and obesity, type 2 diabetes, all-cause mortality, hypertension, CVD incidence were backed up by highly suggestive evidence. Finally, weak evidence was found for colorectal cancer, pancreatic cancer, gastrointestinal cancer, cancer mortality, cardiovascular mortality, chronic kidney disease, CAD, and stroke.

**TABLE 2:** Certainty of evidence of meta-analyses of observational studies linking artificially-sweetened beverages and health outcomes

Health outcomes	Certainty of evidence	Criteria used
-	Strong	∗*P* < 10^−6^; >1000 cases; *P* < 0.05 of the largest study in a meta-analysis; *I*^2^ < 50%; no small study effect^1^; prediction interval excludes the null value; no excess significance bias^2^
Obesity, type 2 diabetes, all-cause mortality, hypertension, CVD incidence	Highly suggestive	∗*P* < 10^−6^; >1000 cases; *P* < 0.05 of the largest study in a meta-analysis
-	Sugestive	∗*P* < 10^−3^; >1000 cases
Colorectal cancer, pancreatic cancer, gastrointestinal cancer, cancer mortality, cardiovascular mortality, chronic kidney disease, CAD, stroke	Weak	∗*P* < 0.05

1 Small study effects is based on the *P* value from the Egger’s regression asymmetry test (*P* ≤ 0.1) where the random-effects model estimate is larger than the point estimate of the largest study.

2 Base on the *P* value (*P* > 0.1) of the excess of significance test using the largest study in a meta-analysis.

∗ *P* indicates the *P* values of the meta-analysis random-effects model.

### Methodological quality of the selected systematic reviews

Results of the evaluation of the methodological quality of selected systematic reviews and meta-analyses by AMSTAR-2 are shown in Table 3. The majority of reports (8 out of 11) followed the PECO elements to describe the aims and methods of the systematic review. Three reports had a well-developed study protocol before conducting the systematic review. Only 1 report justified the selection of study designs eligible for selection purposes. All systematic reviews described partially the search strategy used. The selection of the literature was conducted by a minimum of 2 authors in all reports. Most reports (8 out of 11), extracted data by 2 evaluators. Only 1 report provided a list of excluded studies and their justification. A detailed description of included studies was done in 5 reports. A proper technique to evaluate risk of bias assessments of included studies had partial adherence to the standards recommended by AMSTAR-2 in 10 reports. None of the reports provided details of the sources of funding of each original study selected in different reviews. In 6 reports, authors used appropriate methods for statistical analyses of the meta-analysis. Three reports employed risk of bias assessments in sensitivity analyses. Two reports included comments in the discussion on results of risk of bias assessments. Four reports discussed the potential sources of statistical heterogeneity in the review. Finally, 9 of 11 reports assessed publication bias and discussed their influence in their conclusions.

**TABLE 3:** Methodological quality of selected systematic reviews and meta-analyses using AMSTAR-2

Reference	Outcome variable	AMSTAR-2 items															
		1	2	3	4	5	6	7	8	9	10	11	12	13	14	15	16
Baghavathula et al. [15]	Cardiovascular mortality	Yes	No	No	Partial yes	Yes	Yes	No	Partial yes	Partial yes	No	No	No	Yes	No	Yes	Yes
Jatho et al. [16]	Gastrointestinal cancer	Yes	No	No	Partial yes	Yes	No	No	Partial yes	Partial yes	No	Yes	Yes	No	No	Yes	No
Jatho et al. [16]	Colorectal cancer	Yes	No	No	Partial yes	Yes	No	No	Partial yes	Partial yes	No	Yes	Yes	No	No	Yes	No
Lo et al. [17]	Chronic kidney Disease	Yes	Yes	No	Partial yes	Yes	No	No	Partial yes	Partial yes	No	No	No	No	No	Yes	Yes
Pan et al. [6]	All-cause mortality	No	Partial yes	No	Partial yes	Yes	Yes	Partial yes	Partial yes	Partial yes	No	No	No	No	No	Yes	Yes
Pan et al. [6]	Cancer mortality	No	Partial yes	No	Partial yes	Yes	Yes	Partial yes	Partial yes	Partial yes	No	No	No	No	No	Yes	Yes
Qin et al. [4]	Obesity	Yes	No	No	Partial yes	Yes	Yes	No	Yes	Partial yes	No	Yes	No	No	Yes	Yes	Yes
Qin et al. [4]	Type 2 diabetes	Yes	No	No	Partial yes	Yes	Yes	No	Yes	Partial yes	No	Yes	No	No	Yes	Yes	Yes
Qin et al. [4]	Hypertension	Yes	No	No	Partial yes	Yes	Yes	No	Yes	Partial yes	No	Yes	No	No	Yes	No	Yes
Yin et al. [18]	CVD incidence	No	Yes	Yes	Partial yes	Yes	Yes	Yes	Yes	Partial yes	No	Yes	Yes	Yes	Yes	Yes	Yes
Llaha et al. [19]	Pancreatic cancer	Yes	Yes	No	Partial yes	Yes	Yes	No	Yes	Yes	No	No	No	No	No	No	Yes

### Sensitivity analyses

Main effect sizes of original meta-analyses (RR of Supplemental Table 2) whose outcomes were supported by highly suggestive evidence (Table 2) did not differ in the direction of the effect estimates obtained in our umbrella review (RRs of the random-effects model in Table 1). However, the effect sizes found in the present umbrella review of ASBs were slightly stronger than those reported in the eligible meta-analyses.

## Discussion

In this umbrella review, we graded the certainty of evidence of associations between the consumption of ASBs and 13 health outcomes, evaluating the credibility of the epidemiologic evidence of cohort studies. In our main analyses (Table 1), a high-ASBs intake showed a positive and statistically significant association with 9 outcomes: cardiovascular mortality, chronic kidney disease, all-cause mortality, obesity, type 2 diabetes, hypertension, CVD incidence (including CAD and stroke), and pancreatic cancer. However, after grading the certainty of evidence using an array of statistical tests, we found that only 5 outcomes (obesity, type 2 diabetes, all-cause mortality, hypertension, and CVD incidence) had highly suggestive evidence.

Recent experimental studies in humans have demonstrated rapid, harmful cardiometabolic changes induced by artificial sweeteners [3,20]. Sucralose and saccharin have particularly been associated with impaired glucose tolerance in healthy adults after just 2 wk of daily supplementation [3]. The experimental effects of sucralose on the human gut microbiota have, however, produced inconsistent findings between studies. Some clinical trials reported no effects of sucralose on the relative abundance of intestinal bacteria [21,22], whereas others found that low dosages of daily sucralose (48 mg) during 10 wk produced a 3-fold increase in firmicutes (Blautia coccoides) and a decrease (0.66-fold) in Lactobacillus Acidophilus [20]. These controversial findings could be explained by the different dosages of sucralose used among different studies. A bell-shaped dose-response effect has been suggested to explain the null effects observed in trials with very high doses of sucralose [20]. Furthermore, a growing number of animal studies have suggested that some artificial sweeteners may induce gut wall immune reactivity [23]. LPS is an endotoxin that increases intestinal permeability and stimulates monocyte and macrophage production of inflammatory mediators. It has been suggested that the consumption of artificial sweeteners (stevia, sucralose, saccharin, and acesulfame potassium) increase LPS values [23] and this change would lead to unfavorable immunologic responses. Although ASBs have been historically recommended to prevent weight gain or type 2 diabetes, our umbrella review indicates that ASBs are associated with an increased risk of obesity or type 2 diabetes. Observational studies of this topic may be prone to bias. ASBs consumers could choose these artificial products because they became ill (for example, developed cardiometabolic risk factors) or experienced recent weight gain, which is known as reverse causation effect in epidemiology. Nevertheless, there is only partial support for this undesired bias because fully adjusted multivariate models tend to attenuate (yet not totally eliminate) the increased risk of type 2 diabetes, particularly after adjustments for BMI at baseline or weight change [4]. It is also worth mentioning that meta-analyses tend to include studies with a large amount of covariates in their models (see Supplemental Table 4). On the other hand, there is clinical evidence that ASBs consumption could influence sweet taste receptors and brain communications. For example, in a clinical trial and after 1 y of follow-up, the sweetness threshold was unaltered in a group of overweight participants that consumed ASBs [24]. In contrast, the sweetness threshold decreased in overweight participants who received no-calorie, unsweetened beverages. Further, well-designed clinical trials will be needed to test the impact of ASBs on appetite and metabolic health markers in humans.

Weak observational evidence was observed for other health outcomes: cancer risk at several sites (colorectal, pancreatic, and gastrointestinal), cancer mortality, chronic kidney disease, or cardiovascular mortality. The 2020 World Cancer Report [25] did not consider ASBs or artificial sweeteners as dietary carcinogens; bacterial mechanisms that promote carcinogenesis are still incompletely elucidated [25]. Although there is weak evidence linking ASB consumption and cancer, it may still be wise to follow the precautionary principle until our knowledge improves on how the intestinal microbiota shapes cancer risk.

In our umbrella review, we found that some published systematic reviews of ASBs had serious flaws in the identification of eligible studies (see Table 3). For example, Jatho et al. [16] concluded that the consumption of ASBs was significantly associated with an increased risk of liver cancer (RR, 1.28; 95% CI: 1.03, 1.58). However, the 2 unique original studies, included in their meta-analysis, evaluated the consumption of ASBs plus SSBs [26] or the consumption of SSBs only [27]. Jatho et al. [16] also inappropriately included some studies that considered artificial sweeteners in supplements (tablets and sachets) instead of ASBs, or whose outcome did not match the outcome searched (cancer unrelated to obesity instead of gastrointestinal cancer). Other major deficiencies in the methods followed by authors of systematic reviews and meta-analyses merit a brief discussion. Results obtained with the AMSTAR-2 tool indicate that most authors of systematic reviews are not making use of predefined study protocols, which may increase the risk of making unfounded decisions during all the stages of the systematic review. Furthermore, all reviews did not add information of the sources of funding of the literature reviewed. The Cochrane handbook for systematic reviews of intervention recommends to examine closely the conflicts of interest of lead and corresponding authors, based on information reported in the present or previous publications or even searching in additional databases (Open Payment Database, clinicaltrials.gov) [28]. To prevent or attenuate the undue commercial influence should be a priority among scientists to increase the trustworthiness of scientific research. It has been reported that authors with financial ties with the ASB industry tend to publish more industry favorable results (for example, weight loss) [29]. Nonetheless, some public health researchers have challenged the value for research integrity of reporting authors conflict of interests, because these partial declarations do not avoid the negative undue influence of the private sector [30].

The present study has several limitations. First, randomized controlled trials are better suited to identify causal effects compared with cohort studies. However, trials of the long-term effects of ASBs on the risk of hard end points (such as CVD) are lacking, being unfeasible to conduct because of their high cost and lack of adherence to long-term interventions [31]. Although some trials have investigated early markers of cardiovascular health, they were of short duration, recruited unhealthy participants and some were industry funded [31]. Meta-analyses of randomized controlled trials are the pinnacle of evidence-based medicine. However, they were excluded from our statistical analyses because they contained a low number of studies (see Supplemental Table 4 and eligibility criteria). Unfortunately, all studies identified in our umbrella review (meta-analysis) did not examine how different types of ASB were associated with health outcomes. In a recent prospective study (median follow-up 9 y), acesulfame potassium and sucralose were associated with a higher risk of CAD (HR, 1.40; 95% CI: 1.06, 1.84) and (HR, 1.31; 95% CI: 1.00, 1.71), respectively, whereas only aspartame remained associated with an increased cerebrovascular disease risk (HR, 1.17; 95% CI: 1.03, 1.33) [31]. Third, it must be noted that artificial sweeteners in ASBs account for a partial part of total consumption of artificial sweeteners. In the French cohort study, NutriNet-Santé [31], 37% of participants consumed artificial sweeteners (42.46 mg/d, average). Soft drinks with no added sugar accounted for 53% of total artificial sweeteners. Other vectors were tabletop sweeteners (30%), artificial sweetened yogurts, and cottage cheese (8%), soft drinks with both sugar and artificial sweeteners (3%), others (6%), such as biscuits (cookies), breakfast cereals, etc. Future studies, if feasible, should evaluate the total amount of artificial sweeteners consumed by participants rather than only those from soft drinks with no added sugar. Finally, it must be acknowledged that compared with traditional systematic reviews, umbrella reviews are still in need of standardization among authors [9]. We made decisions to select the eligible reviews based on previous umbrella reviews published in leading biomedical journals [[32], [33], [34], [35], [36]]. Nonetheless, future umbrella reviews should compare how different methods of selection and analyses influence the results of the umbrella review.

In conclusion, the main findings of our umbrella suggest that ASBs could increase the risk of obesity, type 2 diabetes, all-cause mortality, hypertension, and CVD incidence (highly suggestive evidence). In contrast, weak evidence was found with cancer mortality, cancer incidence, CAD, stroke, cardiovascular mortality, or chronic kidney disease. Changes in the gut microbiota or an increased production of inflammatory markers have been suggested as possible mechanisms linking ASBs and health outcomes. Because of the low number of randomized controlled trials published of ASBs, and their methodological issues: short duration of all interventions, the type of comparator groups used in trials (commonly water plus sugar or SSBs, instead of water), and the predominant use of just 2 artificial sweeteners (acefulsame K and aspartame); further experimental work is urgently needed to determine the chronic effects of ASBs on body weight and cardiometabolic risk management.

## Author disclosures

The authors report no conflicts of interest.

## Data availability

Data described in the manuscript and analytic code will be made available upon request to the corresponding author.

## Appendix A Supplementary data

Multimeia component1 Multimeia component1
