# Supplementary material for: Artificially Sweetened Beverages and Health Outcomes: An Umbrella Review
Source: Adv Nutr. 2023 May 13;14(4):710–7. doi: 10.1016/j.advnut.2023.05.010 (PMC10334147; doi:10.1016/j.advnut.2023.05.010)
Supplement: Multimeia component1 [file mmc1.pdf]

Supplementary Material for Publication

Supplementary Table 1. Umbrella Review: search terms and strategy used

**Search terms for PubMed**
#1 ("beverages"[Mesh] OR "drink"[Title/Abstract] OR "drinks"[Title/Abstract] OR "beverage"[Title/Abstract] OR "beverages"[Title/Abstract] OR "soda"[Title/Abstract] OR "pop"[Title/Abstract] OR "sweetened iced tea"[Title/Abstract] OR "fruit punch"[Title/Abstract] OR "cordials"[Title/Abstract] OR "squashes"[Title/Abstract] OR "lemonade"[Title/Abstract])
#2 ("prospective studies"[Mesh] OR "cohort studies"[Mesh] OR "longitudinal studies"[Mesh] OR "prospective"[Title/Abstract] OR "longitudinal"[Title/Abstract] OR "cohort"[Title/Abstract] OR "cohorts"[Title/Abstract] OR "follow-up"[Title/Abstract] OR “followed up"[Title/Abstract])
#3 (#1 AND #2)
#4 (#3 AND "systematic review"[Title/Abstract] OR "meta-analysis"[Title/Abstract]
**Search terms for Embase**
#1 beverage/ or beverage.mp. or beverages.mp. or drink.mp. or drinks.mp. or soda.mp. or pop.mp. or fruitades.mp. or sweetened iced tea.mp. or fruit punch.mp. or cordials.mp. or squashes.mp. or lemonade.mp.
#2 cohort analysis/ or longitudinal study/ or prospective study/ or follow up/ or prospective.mp. or longitudinal.mp. or cohort.mp. or cohorts.mp. or follow-up.mp. or followed up.mp.
#3 (#1 AND #2)
#4 systematic review.mp. or meta-analysis.mp.
(#3 AND #4)
**Search terms for Web of Science**
#1 TS=("drink" OR "drinks" OR "beverage" OR "beverages" OR "soda" OR "pop" OR "sweetened iced tea" OR "fruit punch" OR "cordials" OR "squashes" OR "lemonade")
#2 TS=("prospective" OR "longitudinal" OR "cohort" OR "cohorts" OR "follow-up" OR "followed up")
#3 (#1 AND #2)

#4 TS=("systematic review" OR "meta-analysis")

Supplementary Table 2. Descriptive data of selected systematic reviews and meta-analysis of ASBs and health outcomes

Author and year of publication	PECO (Population, Intervention, Control and Outcome) question	Exposure variable	Outcome variable	Number of studies in each meta-analysis	Number of cohort studies	Number of case-control studies	Main result in each meta-analysis	Source of funding of the systematic review
Baghavathula et al. 2022 (15)	May the consumption of 2 drinks (250 ml=1 drink), or more of ASBs (versus 0 drinks) increase cardiovascular mortality risk in adults?	2 (o more) drinks versus 0	Cardiovascular mortality	4 reported, 3 met inclusion criteria	3	0	RR 1.33; 95% CI 1.12 to 1.55	No research funding
Jatho et al. 2021 (16)	May the highest consumption of ASBs (versus no or low) increase gastrointestinal cancer risk in adults?	The highest consumption versus no or low	Gastro-intestinal cancer	32 reported, 8 met inclusion criteria	7	1	RR 1.02; 95% CI 0.92 to 1.14	Unreported
Jatho et al. 2021 (16)	May the highest consumption of ASBs (versus no or low) increase colorectal cancer risk in adults?	The highest consumption versus no or low	Colorectal cancer	9 reported, 4 met inclusion criteria	4	0	RR 0.98; 95% CI 0.79 to 1.23	Unreported
Lo et al. 2021 (17)	May the highest consumption of ASBs (versus the lowest) increase Chronic Kidney Disease risk in adults?	The highest consumption versus no or low	Chronic Kidney Disease	3 reported, 2 met inclusion criteria	1	1	RR 1.40; 95% CI 0.65 to 3.02	Shuang Ho Hospital, Taipei Medical University and Ministry of Science and Technology

Pan et al. 2021 (6)	May a higher consumption of ASBs increase all-cause mortality risk in adults?	Per 250 ml/day increase of ASBs	All-cause mortality	7 reported, 7 met inclusion criteria	7	0	RR 1.04; 95% CI 1.00 to 1.09	Gansu Hospital
Pan et al. 2021 (6)	May a higher consumption of ASBs increase cancer mortality risk in adults?	Per 250 ml/day increase of ASBs	Cancer mortality	3 reported, 3 met inclusion criteria	3	0	RR 1.01; 95% CI 0.98 to 1.05	Gansu Hospital
Qin et al. 2020 (4)	May a higher consumption of ASBs increase all-cause mortality risk in adults?	Per 250 ml/day increase of ASBs	Obesity	5 reported, 4 met inclusion criteria	4	0	RR 1.21; 95% CI 1.09 to 1.35	Foundations (National China, Guangdong and Shenzhen)
Qin et al. 2020 (4)	May a higher consumption of ASBs increase Type 2 diabetes risk in adults?	Per 250 ml/day increase of ASBs	Type II Diabetes	13 reported, 11 met inclusion criteria	11	0	RR 1.15; 95% CI 1.05 a 1.26	Foundations (National China, Guangdong and Shenzhen)
Qin et al. 2020 (4)	May a higher consumption of ASBs increase hypertension risk in adults?	Per 250 ml/day increase of ASBs	Hypertension	5 reported, 4 met inclusion criteria	4	0	RR 1.08; 95% CI 1.06 a 1.10	Foundations (National China, Guangdong and Shenzhen)
Yin et al. 2021 (18)	May a higher consumption of ASBs increase cardiovascular disease risk in adults?	Per 250 ml/day increase of ASBs	Cardiovascular Disease (incidence)	8 reported, 8 met inclusion criteria	8	0	RR 1.07; 95% CI 1.05 a 1.10	National Program of Research and Development y
Llaha et al. 2021 (19)	May the highest consumption of ASBs (versus the lowest) increase pancreatic cancer risk in adults?	The highest consumption versus lowest	Pancreatic cancer	5 reported, 5 met inclusion criteria	3	2	RR 1.07; 95% CI 0.77 a 1.48	National health Institute Carlos III

Supplementary Table 3. Original studies excluded (and reasons) of each selected systematic review and meta-analysis of ASBs and health outcomes

**Systematic Review: First author and year of publication**	**Health outcome reported**	**Excluded studies (First author and year of publication, outcome) and reasons**
Baghavathula et al. 2022 (15)	Cardiovascular mortality	Ramne et al. 2019 (Cardiovascular mortality): The exposure variable was Sugar-Sweetened Beverages (SSBs)
Jatho et al. 2021 (16)	Gastro-intestinal cancer	Lagergren et al. 2006 (Esophageal adrenocarcinoma): The exposure variable was SSBs plus ASBs<br>Lagergren et al. 2006 (Gastric Cancer): The exposure variable was SSBs plus ASBs<br>Larsson et al. 2006 (Pancreatic cancer): The exposure variable was SSBs<br>Mayne et al. 2006 (Oesophageal AC): The exposure variable was SSBs plus ASBs<br>Mayne et al. 2006 (Oesophageal SCC): The exposure variable was SSBs plus ASBs<br>Mayne et al. 2006 (Gastric Cardia): The exposure variable was SSBs plus ASBs<br>Mayne et al. 2006 (Gastric non-cardia): The exposure variable was SSBs plus ASBs<br>Gallus et al. 2007 (Colorectal colon): The exposure variable included teaspoons/week, saccharin, and other sweeteners, expressed in sachets or tablets/week<br>Gallus et al. 2007 (Colorectal rectum): The exposure variable included teaspoons/week, saccharin, and other sweeteners, expressed in sachets or tablets/week

		Gallus et al. 2007 (Oesophageal): The exposure variable included teaspoons/week, saccharin, and other sweeteners, expressed in sachets or tablets/week<br>Ibiebele et al. 2008 (Oesophageal AC): The exposure variable was SSBs plus ASBs<br>Ibiebele et al. 2008 (Oesophageal AEG): The exposure variable was SSBs plus ASBs<br>Ibiebele et al. 2008 (Oesophageal SCC): The exposure variable was SSBs plus ASBs<br>Bossetti et al. 2009 (Gastric cancer): The exposure variable included teaspoons/week, saccharin, and other sweeteners, expressed in sachets or tablets/week<br>Bossetti et al. 2009 (Pancreatic cancer): The exposure variable included teaspoons/week, saccharin, and other sweeteners, expressed in sachets or tablets/week<br>Gallus et al. 2007 (Pancreatic cancer): The exposure variable included teaspoons/week, saccharin, and other sweeteners, expressed in sachets or tablets/week<br>Mahfouz et al. 2014 (Colorectal cancer): The exposure variable was poorly defined: Ex. artificial sweeteners (unclear if they included food supplements plus beverages or not)<br>Stepien et al. 2016 (Liver cancer-Men): The exposure variable was SSBs plus ASBs<br>Stepien et al. 2016 (Liver cancer-Women): The exposure variable was SSBs plus ASBs<br>Li et al. 2017 (Oesophageal cancer-Men): The exposure variable was SSBs<br>Li et al. 2017 (Oesophageal cancer-Women): The exposure variable was SSBs<br>Li et al. 2017 (Gastric cancer): The exposure variable was SSBs<br>Bassett et al. 2020: The outcome variable included a non-specific cancer site, cancers unrelated to obesity<br>Luo et al. 2019 (Liver cancer): The exposure variable was SSBs

Jatho et al. 2021 (16)	Colorectal cancer	Khan et al. 2004 (Colorectal-Men): The exposure variable was SSBs plus ASBs plus juices<br>Khan et al. 2004 (Colorectal-Women): The exposure variable was SSBs plus ASBs plus juices<br>Gallus et al. 2007 (Colon): The exposure variable included teaspoons/week, saccharin, and other sweeteners, expressed in sachets or tablets/week<br>Gallus et al. 2007 (Rectum): The exposure variable included teaspoons/week, saccharin, and other sweeteners, expressed in sachets or tablets/week<br>Mahfouz et al. 2014 (Colorectal): The exposure variable was poorly defined: Ex. artificial sweeteners (unclear if they included food supplements plus beverages or not)
Lo et al. 2021 (17)	Chronic Kidney Disease	Bomback et al. 2010: The exposure variable was SSBs
Qin et al. 2020 (4)	Obesity	Duffey et al. 2010: The reference group was ASBs consumers (exposure group: non-consumers of ASBs)
Qin et al. 2020 (4)	Type 2 Diabetes	Ma et al. 2016: The outcome variable was defined as prediabetes, instead Type 2 diabetes<br>Duffey et al. 2010: The reference group was ASBs consumers (exposure group: non-consumers of ASBs)
Qin et al. 2020 (4)	Hypertension	Duffey et al. 2010: The reference group was ASBs consumers (exposure group: non-consumers of ASBs)

Supplementary Table 4. Systematic reviews of randomized controlled trials (intervention arm: ASBs) versus (control arms) on health outcomes

Author	Journal	Sample	Health outcome	Number of RCTs	Control group	Duration	Effects
Brown et al. 2010	Int J Pediatr Obes	0-18 years	Weight loss or BMI differences	3 trials			
				Knopp et al. 1976	2.7 gr/day Aspartame versus placebo	13 weeks	**No significant differences in weight loss**
				Ebbeling et al. 2006	Replacing SSBs with ASBs, versus control	25 weeks	**No significant difference in BMI** between those in intervention (replacing SSBs with ASBs) vs. control group except among heaviest subjects
				Williams et al. 2007	ASBs versus SSB	12 weeks	**No significant difference in BMI** between those permitted sugar-

							sweetened soda vs. those only artificially-sweetened soda
Mehat et al. 2022	Adv in Nutr	Adults	Energy intake and glucose levels	3 trials<br><br>Bonnet et al. 2018<br><br><br><br><br><br><br><br>Kim et al. 2020	660 ml/day carbonated water versus carbonated water with aspartame and acefulsame K<br><br>600 ml/day carbonated water versus ASBs with aspartame and acefulsame K	2 x 12 weeks Cross-over<br><br><br><br><br>2 x 2 weeks Cross-over	Energy intake (24 h)<br><br>**Asp/Ace-K < water (*P* = 0.002)**<br><br><br><br><br>Blood glucose (60 min)<br><br>**No significant difference between Asp/Ace-K when compared with water and sugar controls**

				Van Wymelbeke et al. 2004	Mineral water with sucrose with orange or raspberry versus ASBs with with aspartame, saccharin and acefulsame K	2 x 10 weeks Cross-over	Energy intake (48 h)<br><br>**Asp/Ace-K < sugar (*P* = 0.004)**
Espinosa et al. 2023	The lancet global health	Children and adolescents	BMI	5 trials	Non-nutritive sweeteners (25–2400 mg/day, from food and beverages versus intake of sugar from food and beverages	Median follow-up 19 weeks	Less BMI gain (standardised mean difference –0·42 kg/m^2 [95% CI –0·79 to –0·06]; I^2=89%) compared with the intake of sugar from food and beverages.<br><br>Removing studies with potential conflicts of

							interest attenuated the estimates.<br><br>**No randomised controlled trials tested beverages containing non-nutritive sweeteners versus water.**

References:

Ramne S, Alves Dias J, González-Padilla E, Olsson K, Lindahl B, Engström G, Ericson U, Johansson I, Sonestedt E. Association between added sugar intake and mortality is nonlinear and dependent on sugar source in 2 Swedish population-based prospective cohorts. *Am J Clin Nutr.* 2019;109(2):411-23.

Lagergren J, Viklund P, Jansson C. Carbonated soft drinks and risk of esophageal adenocarcinoma: a population-based case-control study. *J Natl Cancer Inst.* 2006;98(16):1158-61.

Larsson SC, Bergkvist L, Wolk A. Consumption of sugar and sugar-sweetened foods and the risk of pancreatic cancer in a prospective study. *Am J Clin Nutr.* 2006;84:1171-6.

Mayne ST, Risch HA, Dubrow R, Chow WH, Gammon MD, Vaughan TL, Borchardt L, Schoenberg JB, Stanford JL, West AB. Carbonated soft drink consumption and risk of esophageal adenocarcinoma. *J Natl Cancer Inst.* 2006;98;72-5.

Gallus S, Scotti L, Negri E, Talamini R, Franceschi S, Montella M, Giacosa A, Dal Maso L, La Vecchia C. Artificial sweeteners and cancer risk in a network of case–control studies. *Ann Oncol.* 2007;18,40-4.

Ibiebele TI, Hughes MC, O'Rourke P, Webb PM, Whiteman DC; Australian Cancer Study. Cancers of the esophagus and carbonated beverage consumption: a population-based case-control study. *Cancer Causes Control.* 2008;19, 577-84.

Bosetti C, Gallus S, Talamini R, Montella M, Franceschi S, Negri E, La Vecchia C. Artificial sweeteners and the risk of gastric, pancreatic, and endometrial cancers in Italy. *Cancer Epidemiol Biomarkers Prev.* 2009;18(8):2235-8.

Mahfouz EM, Sadek RR, Abdel-Latief WM, Mosallem FA, Hassan EE. The role of dietary and lifestyle factors in the development of colorectal cancer: case control study in Minia, Egypt. *Cent Eur J Public Health.* 2014;22(4):215-22.

Stepien M, Duarte-Salles T, Fedirko V, Trichopoulou A, Lagiou P, Bamia C, Overvad K, Tjønneland A, Hansen L, Boutron-Ruault MC, et al. Consumption of soft drinks and juices and risk of liver and biliary tract cancers in a European cohort. *Eur J Nutr*. 2016;55(1):7-20.

Li N, Petrick JL, Steck SE, Bradshaw PT, McClain KM, Niehoff NM, Engel LS, Shaheen NJ, Risch HA, Vaughan TL. A pooled analysis of dietary sugar/carbohydrate intake and esophageal and gastric cardia adenocarcinoma incidence and survival in the USA. *Int J Epidemiol.* 2017;46(6):1836-46.

Bassett JK, Milne RL, English DR, Giles GG, Hodge AM. Consumption of sugar-sweetened and artificially sweetened soft drinks and risk of cancers not related to obesity. *Int J Cancer.* 2020;146(12):3329-34.

Luo X, Sui J, Yang W, Sun Q, Ma Y, Simon TG, Liang G, Meyerhardt JA, Chan AT, Giovannucci EL, et al. Type 2 Diabetes prevention diet and hepatocellular carcinoma risk in US men and women. *Am J Gastroenterol.* 2019;114(12):1870-7.

Khan MM, Goto R, Kobayashi K, Suzumura S, Nagata Y, Sonoda T, Sakauchi F, Washio M, Mori M. Dietary habits and cancer mortality among middle aged and older Japanese living in hokkaido, Japan by cancer site and sex. *Asian Pac J Cancer Prev.* 2004;5(1):58-65.

Bomback AS, Derebail VK, Shoham DA, Anderson CA, Steffen LM, Rosamond WD, Kshirsagar AV. Sugar-sweetened soda consumption, hyperuricemia, and kidney disease. *Kidney Int.* 2010;77(7):609-16.

Duffey KJ, Gordon-Larsen P, Steffen LM, Jacobs DR Jr, Popkin BM. Drinking caloric beverages increases the risk of adverse cardiometabolic outcomes in the Coronary Artery Risk Development in Young Adults (CARDIA) Study. *Am J Clin Nutr*. 2010;92(4):954-9.

Ma J, Jacques PF, Meigs JB, Fox CS, Rogers GT, Smith CE, Hruby A, Saltzman E, McKeown NM. Sugar-sweetened beverage but not diet soda consumption is

positively associated with progression of insulin resistance and prediabetes. *J Nutr.* 2016;146(12):2544-50.

Qin P, Li Q, Zhao Y, Chen Q, Sun X, Liu Y, Li H, Wang T, Chen X, Zhou Q, et al. Sugar and artificially sweetened beverages and risk of obesity, type 2 diabetes mellitus, hypertension, and all-cause mortality: a dose-response meta-analysis of prospective cohort studies. *Eur J Epidemiol.* 2020;35(7):655-71.

Baghavathula AS, Rahmani J, Vidyasagar K, Tesfaye W, Khubchandani J. Sweetened beverage consumption and risk of cardiovascular mortality: A systematic review and meta-analysis. *Diabetes Metab Syndr.* 2022;16(4):102462.

Jatho A, Cambia JM, Myung SK. Consumption of artificially sweetened soft drinks and risk of gastrointestinal cancer: a meta-analysis of observational studies. *Public Health Nutr.* 2021;24(18):6122-36.

Lo WC, Ou SH, Chou CL, Chen JS, Wu MY, Wu MS. Sugar- and artificially-sweetened beverages and the risks of chronic kidney disease: a systematic review and dose-response meta-analysis. *J Nephrol.* 2021;34(6):1791-804.

Brown RJ, de Banate MA, Rother KI. Artificial sweeteners: a systematic review of metabolic effects in youth. *Int J Pediatr Obes.* 2010; 5(4):305-12.

Mehat K, Chen Y, Corpe CP. The combined effects of aspartame and acesulfame-K blends on appetite: A systematic review and meta-analysis of randomized clinical trials. *Adv Nutr.* 2022;13(6):2329-40.

Espinosa A, Mendoza K, Laviada-Molina H, Rangel-Méndez JA, Molina-Segui F, Sun Q, Tobias DK, Willett WC, Mattei J. Effects of non-nutritive sweeteners on the BMI of children and adolescents: a systematic review and meta-analysis of randomised controlled trials and prospective cohort studies. *Lancet Glob Health.* 2023;11 Suppl 1:S8.
